# Nitazoxanide alleviates collagen-induced arthritis by inhibiting STAT3-dependent Th17 cell differentiation and inflammatory cytokines production

**DOI:** 10.3389/fphar.2026.1877306

**Published:** 2026-09-23

**Authors:** Changhong Li, Xiaoli Chen, Yijun Han, Yan Niu, Huijie Leng, Jinxia Zhao

**Affiliations:** 1 Department of Rheumatology and Immunology, Peking University Third Hospital, Beijing, China; 2 Department of Medicinal Chemistry, School of Pharmaceutical Sciences, Peking University Health Science Center, Beijing, China; 3 Department of Orthopaedics, Peking University Third Hospital, Beijing, China

**Keywords:** inflammatory cytokines, nitazoxanide, rheumatoid arthritis, stat3, Th17 cell

## Abstract

Nitazoxanide (NTZ), an FDA-approved anti-parasitic drug, has shown anti-inflammatory effects in rheumatoid arthritis (RA) fibroblast-like synoviocytes, yet its impact on immune cell function and its potential synergy with methotrexate (MTX) remains unclear. In this study, using a murine collagen-induced arthritis (CIA) model, we demonstrated that NTZ synergizes with MTX to delay arthritis onset and ameliorate joint pathology, accompanied by reduced inflammatory cell infiltration, synovial pannus formation, and bone destruction. Moreover, NTZ significantly suppressed inflammatory cytokine production in both murine splenocytes and human peripheral blood mononuclear cells from healthy donors and RA patients. *In vivo* findings from the CIA model further demonstrated that NTZ suppressed synovial IL-6 and pSTAT3 expression, as well as Th17 cell differentiation. And these effects collectively contributed to the improvement in arthritis score. Mechanistically, NTZ markedly inhibited the STAT3 signaling pathway in immune cells upon IL-6 or anti-CD3 stimulation. Additionally, it potently suppressed osteoclast differentiation of monocytes derived from RA patients. Taken together, this study provides a proof-of-concept for NTZ’s immunomodulatory activity in RA, yet the data remain insufficient for therapeutic claims and only lay the groundwork for further studies.

## Introduction

As a prevalent and aggressive autoimmune disorder, rheumatoid arthritis (RA) involves inflammatory cell invasion and abnormal growth in the synovium. This process ultimately leads to the erosion of nearby cartilage and bone, causing progressive joint damage, functional limitations, and a significant decline in patient’s quality of life (McInnes and Schett, 2017; Smolen et al., 2016).

While the exact pathogenesis of RA remains incompletely elucidated, it is established that its development is driven by a complex interplay of factors. Among them, inflammatory cytokines like IL-1β, TNF-α and IL-6, secreted by inflammatory cells within affected joints, are crucial mediators (McInnes and Schett, 2017). They perpetuate local inflammation and instigate bone erosion, partly by stimulating synovial fibroblasts to release destructive enzymes such as matrix metalloproteinases (MMPs) (Chen et al., 2019). Furthermore, Th17 cells and their signature cytokine, IL-17A, contribute to the autoimmune pathology by breaking the immune balance and activating pathway like STAT3 (Komatsu et al., 2014; Mo et al., 2024; Lee et al., 2013; Khokhar et al., 2025). Despite the proven efficacy of Janus kinase inhibitors in treating RA, uncertainties persist concerning their safety and overall risk-benefit balance (Wang L. et al., 2025). Hence, the development of safer and more effective therapeutic agents remains an urgent priority.

Nitazoxanide (NTZ) is an FDA-approved thiazolide anti-parasitic agent (White, 2004). In addition to its conventional anti-parasitic applications, NTZ has demonstrated broad-spectrum activity against a variety of pathogens and neoplastic diseases (Cui et al., 2024; Lü et al., 2021), which has spurred interest in its potential for drug repurposing, including for COVID-19 (Kelleni, 2023). Mechanistic studies indicate that NTZ modulates multiple signaling pathways, such as Wnt/β-catenin (Qu et al., 2018), c-Myc (Fan-Minogue et al., 2013), mTORC1 (Lam et al., 2012; Kim et al., 2025) and STAT3 (Lü et al., 2021). Notably, our recent work identified NTZ as a dual inhibitor of STAT3 and NF-κB, showing that it can alleviate inflammation and bone erosion in a mouse model of collagen-induced arthritis (CIA) (Li et al., 2024), thereby underscoring its therapeutic potential for inflammatory diseases. However, that study focused exclusively on the effects of NTZ on RA fibroblast-like synoviocytes (FLS), leaving its impact on immune cells unaddressed. To a large extent, this part of the study represents a logical continuation of our previous research. Moreover, although methotrexate (MTX) serves as a first-line therapy for RA, its monotherapy often leads to suboptimal clinical responses, necessitating combination with other anti-rheumatic drugs, including the addition of conventional synthetic disease-modifying antirheumatic drug (DMARDs) or biologic DMARDs (Duquesne et al., 2023; Fraenkel et al., 2021). And this aligns with the medication strategy recommended by European Alliance of Associations for Rheumatology (EULAR) after the failure of initial MTX treatment (Smolen et al., 2026). Therefore, a preliminary evaluation of the therapeutic efficacy of the MTX–NTZ combination in CIA, along with an exploration of the effects and mechanisms of NTZ on immune cells, may provide a theoretical basis for potential future therapy regimens.

## Materials and methods

### Reagents

Nitazoxanide was purchased from Selleck Chemicals (Shanghai, China), and the compound purity was more than 99%. Recombinant human RANKL and M-CSF were bought from Peprotech (Rocky Hill, NJ, United States). All antibodies for western blot (STAT3, p-STAT3, and β-actin) were supplied by Cell Signaling Technology (Beverly, MA, United States). HRP-conjugated secondary antibodies were obtained from.

ZSGB Bioscience (Beijing, China). Bovine type II collagen (CII) and and Freund’s adjuvant were provided by Chondrex (Seattle, WA, United States). Tartrate resistant acid phosphatase (TRAP) staining kit was bought from Sigma-Aldrich. The details regarding the antibodies and recombinant cytokines utilized in flow cytometry, osteoclast and Th17 cell differentiation can be found in Supplementary Table S1. All ELISA kits were purchased from Multi Science (Hangzhou, China).

### Animal

Six to 8-week male DBA/1 J mice were purchased from Huafukang Bioscience Company (Beijing, China). The mice were housed under specific pathogen-free conditions at the Peking University Third Hospital with *ad libitum* access to water and mouse chow. All animal experimental procedures were performed in accordance with the Guide for the Care and Use Laboratory Animals and the related ethical regulations of Peking University Third Hospital (2023-014-02).

### CIA induction, arthritis assessment and drug administration

The method for CIA induction was as detailed in our prior work (Li et al., 2024). Briefly, male DBA/1J mice received a primary intradermal immunization at the tail base on day 0 with 100 µg of CII emulsified in complete Freund’s adjuvant (CFA). On day 21, mice were anesthetized with isoflurane and given a booster injection of 100 µg CII in incomplete Freund’s adjuvant (IFA). Then, mice were monitored daily for arthritis severity. A standardized scoring system (0–4) was used to grade inflammation in each paw: 0, normal; 1, erythema and mild swelling of one or several digits; 2, erythema and mild swelling extending from the ankle to the midfoot; 3, erythema and moderate swelling extending to the metatarsals; 4, erythema and severe swelling of the entire paw with ankylosis. For therapeutic intervention, mice received oral gavage administrations from day 21 with one of the following: vehicle only (0.5% carboxymethylcellulose (CMC)), MTX (1.5 mg/kg in 0.5% CMC), NTZ (100 mg/kg in 0.5% CMC) or a combination of MTX and NTZ (1.5 mg/kg + 100 mg/kg in 0.5% CMC). MTX was administered three times per week, while all other administrations were given once daily. The NTZ dosage was mainly determined based on the preliminary findings from our previous studies and those of others (Li et al., 2021; Li et al., 2024; El-Kowrany et al., 2019; Wang X. et al., 2025).

### Histopathology and micro-CT

For histological analysis, mice were euthanized, and the right paws were collected. The samples were fixed in 4% paraformaldehyde, decalcified in EDTA, and then embedded in paraffin and sectioned. The sections were stained with hematoxylin and eosin (H&E) and assessed by investigators blinded to the experimental groups. Pathological features, including synovitis, pannus formation, and bone/cartilage destruction, were evaluated according to a previously established method (Tang et al., 2011). Additionally, Safranin O staining was performed to evaluate cartilage proteoglycan content. The expression of IL-6, TNF-α, and pSTAT3 in synovium was detected by immunohistochemistry. The left paws were subjected to high-resolution micro-computed tomography (micro-CT) scanning using an Inveon scanner (Siemens, United States) at a spatial resolution of 15 μm, with X-ray settings of 80 kV and 500 µA. The acquired images were reconstructed and analyzed using the Inveon Research Workplace software (Siemens, United States).

### Murine T cell isolation and cell culture

Splenocytes were aseptically isolated from the spleens of normal C57BL/6 or collagen-induced arthritis (CIA) mice and mechanically dissociated into single-cell suspensions. Erythrocytes were lysed using a commercial lysis buffer (Applygen, Beijing, China). Naïve CD4^+^ T cells were subsequently purified from the splenocytes using the MojoSort™ Mouse CD4 Naïve T Cell Isolation Kit (Biolegend) according to the manufacturer’s instructions. The isolated T cells were activated and expanded in T cell medium (RPMI-1640 supplemented with 10% fetal bovine serum, 1% penicillin-streptomycin, and 50 μM β-mercaptoethanol) using plate-bound anti-CD3 and anti-CD28 antibodies, as previously described (Zani et al., 2023). For Th17 polarization, the cells were cultured in the presence of 1 ng/mL TGF-β1, 20 ng/mL IL-6, 10 ng/mL murine IL-1β, and 20 ng/mL murine IL-23. In most *in vitro* experiments designed to evaluate the effects of NTZ, splenic CD4^+^ T cells were pre-treated with NTZ for 2 h prior to stimulation under Th17-polarizing conditions. Cells were harvested on day 3 for analysis by flow cytometry. For western blot analysis of pSTAT3 levels, CD4^+^ T cells were stimulated with either IL-6 for 30 min or with anti-CD3 and anti-CD28 antibodies for 12 h, in the presence of varying concentrations of NTZ.

### Isolation of human peripheral blood mononuclear cells (PBMCs) and cell stimulation

Human peripheral blood samples were collected from patients diagnosed with RA (n = 3, all were female. Demographic and clinical characteristics summarized in Supplementary Table S2). Three healthy volunteers were enrolled as controls. All participants provided written informed consent following the principles of the Declaration of Helsinki. The study protocol was approved by the Institutional Review Board of Peking University Third Hospital under permit number 2024-540-02. PBMCs were isolated from the buffy coat by density gradient centrifugation using Ficoll–Hypaque. Isolated PBMCs were pretreated with NTZ for 2 h, followed by stimulation with anti-CD3 antibody for 3 days. For western blot analysis of pSTAT3 levels, PBMCs were stimulated with anti-CD3 antibodies for 12 h, in the presence of varying concentrations of NTZ.

### Human *in vitro* osteoclastogenesis formation and TRAP staining

CD14^+^ monocytes were isolated from PBMCs using magnetic-activated cell sorting (MACS; Miltenyi Biotec, Germany), achieving a purity exceeding 99%. The isolated monocytes were then cultured for 3 days in medium supplemented with M-CSF (30 ng/mL) to promote differentiation into pre-osteoclasts. Subsequently, these pre-osteoclasts were further cultured for 7 days in the presence of M-CSF (30 ng/mL), RANKL (50 ng/mL) and NTZ to induce osteoclast formation. On day 3 of this 7-day differentiation period, the medium was replaced with fresh medium containing the same concentrations of M-CSF, RANKL and NTZ. Osteoclast formation was assessed by TRAP staining (Sigma-Aldrich), performed according to the manufacturer’s instructions. Multinucleated cells (containing three or more nuclei) that stained positive for TRAP were identified as osteoclasts and quantified under a light microscope.

### Enzyme-linked immunosorbent assay (ELISA)

The concentrations of cytokines and immunoglobulins were quantified using commercial ELISA kits. Specifically, levels of IL-6, IL-1β, TNF-α, and IgG were measured in mouse samples, while IL-6 and IL-17 were assessed in human samples. Color development was achieved using HRP-conjugated avidin, and the absorbance of each well was read at 450 nm with a reference wavelength of 570 nm on an ELISA microplate reader.

### Flow cytometry

Single-cell suspensions obtained from *in vitro* cultures or *in vivo* mouse experiments were stained for surface markers in PBS at 4 °C for 30 min. For intracellular IL-17A detection, cells were stimulated with Cell Stimulation Cocktail (PMA & ionomycin, BioLegend) in the presence of Brefeldin A (BioLegend) for 4 h, followed by staining using the FOXP3/Transcription Factor Staining Buffer Set (eBioscience). All fluorescently labeled antibodies were used at a 1:100 dilution. Cell samples were analyzed on a CytoFLEX flow cytometer (Beckman Coulter), and data processing was performed using FlowJo software (Tree Star). Gating strategy for the flow cytometric analyses was shown in Supplementary Figure S1.

### Western blotting

Cells were lysed with Radio-immunoprecipitation Assay (RIPA) lysis buffer (Apllygen, Beijing, China) supplemented with phosphatase and protease inhibitors. Protein concentrations were determined using the Bradford assay. Subsequently, 40 μg of protein per lane was separated by 10% SDS-PAGE and transferred onto a polyvinylidene fluoride (PVDF) membrane (Immobilon-P, MA, United States). After blocking with 5% non-fat milk for 2 h, the membranes were incubated overnight at 4 °C on a rotary shaker with the following primary antibodies: STAT3 (1:1000), p-STAT3 (1:1000), and β-actin (1:5000). The membranes were then probed with horseradish peroxidase (HRP)-conjugated secondary antibodies. Protein bands were visualized using an enhanced chemiluminescence (ECL) detection kit (Apllygen, Beijing, China), and band intensities were quantified with ImageJ software.

### Statistical analysis

Quantitative data were represented as means ± SD. All data were tested for normality using the Shapiro-Wilk test and for homogeneity of variances using Levene’s test. The analyses were performed with GraphPad Prism software (version 10 for Windows). For two or more groups, one-way ANOVA or two-way ANOVA followed by Tukey’s or Sidak’s test was used. Fisher’s exact test was used to compare the rates between the two groups. Differences with a p-value of less than 0.05 were deemed statistically significant.

## Results

### Combination therapy with MTX and NTZ prevented the onset of CIA

MTX, an anchor drug for RA, is recommended as first-line therapy by clinical guidelines in numerous countries (Smolen et al., 2023; Cubberley and Maharaj, 2025). However, around 30%–40% of patients with RA respond inadequately to MTX monotherapy, requiring MTX based combination regimens for effective disease control (Duquesne et al., 2023). This study therefore evaluated the effect of NTZ combined with MTX on ameliorating joint inflammation in mice (Figure 1A). As shown in Figure 1B, although either MTX or NTZ alone partially suppressed arthritis score, their overall efficacy was lower than that of the MTX–NTZ combination group. The combination therapy also resulted in a significantly lower cumulative incidence of arthritis compared with the CIA model group and the monotherapy groups (Figure 1C). Histopathological assessment further revealed that the MTX–NTZ combination markedly reduced intra-articular inflammatory cell infiltration, cartilage destruction, and synovial pannus formation relative to the CIA group (Figures 1D,E). These findings demonstrate the superior efficacy of the MTX–NTZ combination over monotherapy, providing a rationale for its potential clinical application.

**FIGURE 1:**
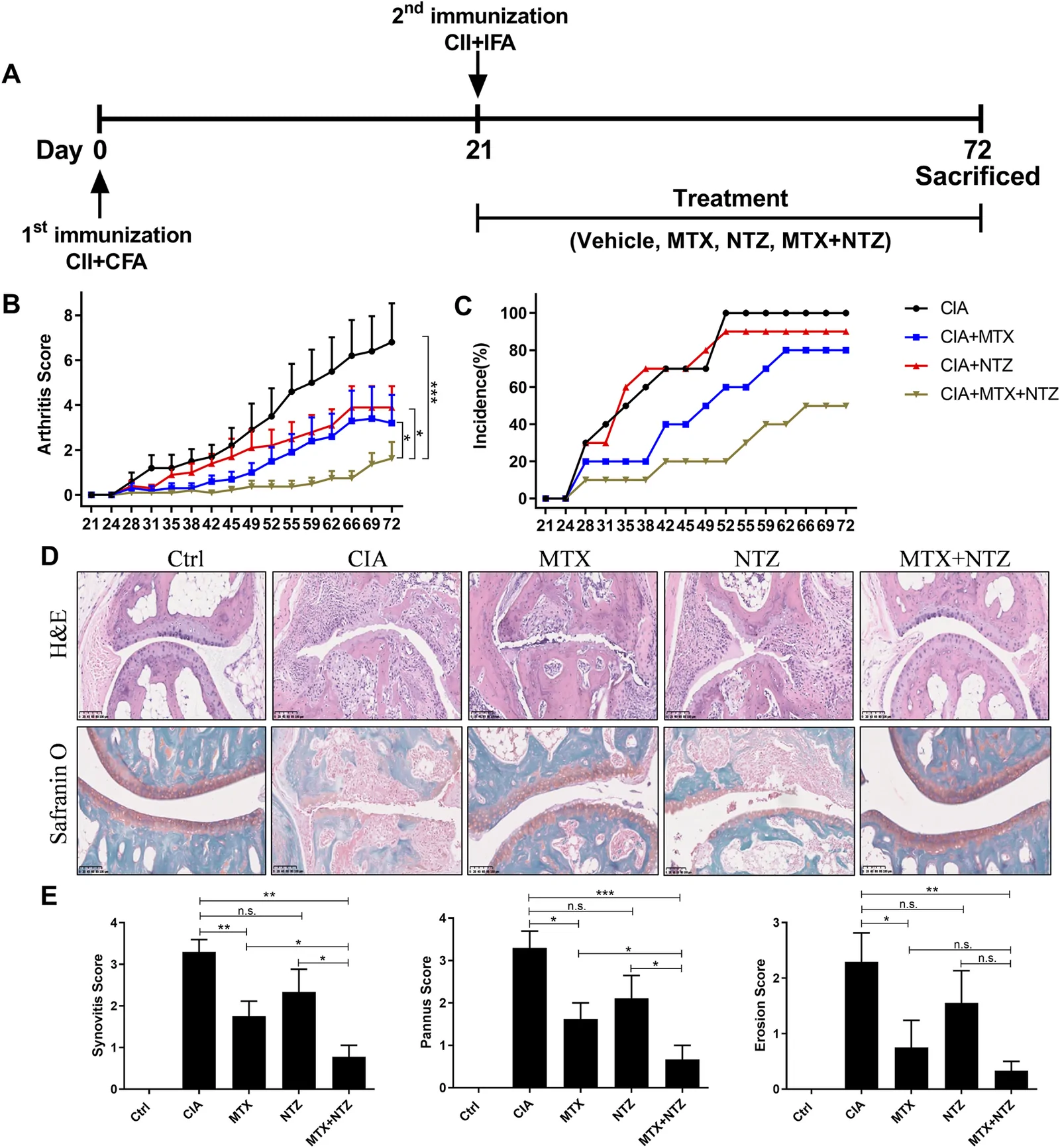
Combination therapy of NTZ and MTX alleviated disease progression in collagen-induced arthritis. **(A)** A graphic scheme of CIA induction and drug administration. **(B)** Arthritis scores for each treatment group assessed at 3∼4-day intervals following the secondary immunization. Values represent mean ± SD (n = 10 per group). Two-way repeated measures ANOVA with Sidak’s multiple comparison. **(C)** Arthritis incidence across the designated groups. Fisher’s exact test. **(D,E)** Representative histological sections of joints stained with H&E and Safranin O, along with scoring of synovitis, pannus formation, and bone erosion. Scale bar = 100 µm. **(E)** One-way ANOVA with Tukey’s multiple comparison. *p < 0.05, **p < 0.01, ***p < 0.001, n.s. not significant.

### MTX–NTZ combination markedly attenuated bone destruction in CIA

Bone destruction is a hallmark pathological feature of RA. To evaluate the effect of combination therapy on global bone destruction in CIA mouse paws, micro-CT scanning and reconstruction were performed. The CIA group showed more severe bone destruction than the control group (Figure 2A). MTX provided greater bone protection than NTZ alone, though both monotherapies were less effective than the MTX-NTZ combination (Figure 2A). Subsequent microstructural analysis of subchondral bone in the proximal tibia revealed that the protective effects of each treatment on trabecular bone correlated with the bone preservation outcomes (Figure 2B). Trabecular microstructural analysis indicated that MTX and MTX-NTZ combination therapy significantly reversed CIA-induced trabecular bone loss. Although NTZ monotherapy did not reach statistical significance, it showed a protective trend, with increased trabecular bone volume (p = 0.054), number (p = 0.138), and thickness (p = 0.081), along with reduced trabecular separation (p = 0.188) compared to the CIA group (Figure 2C).

**FIGURE 2:**
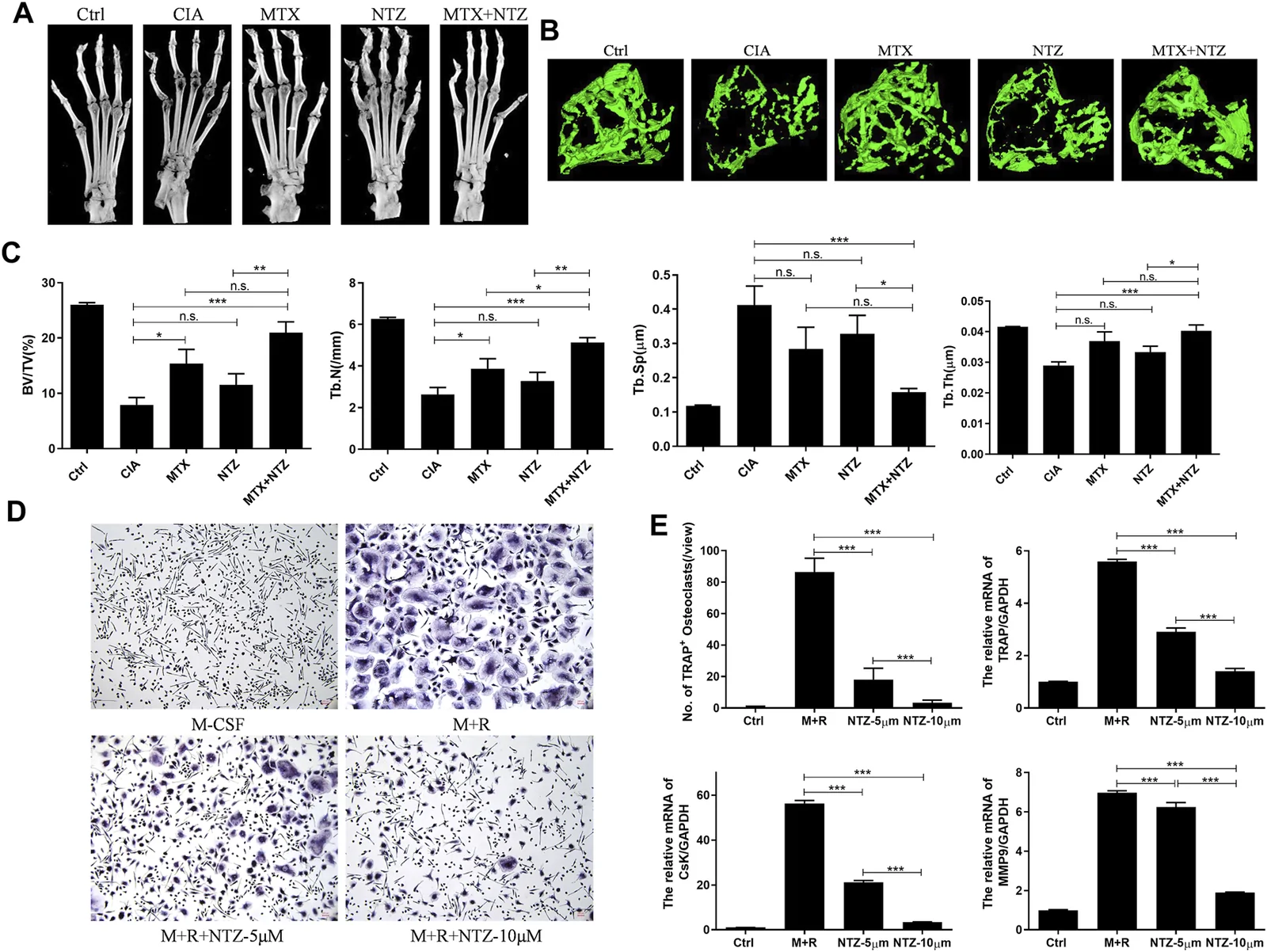
NTZ combined with MTX markedly inhibited bone erosion in the CIA model. **(A)** Representative 3D micro-CT reconstructions of mouse hind paws from each group. **(B)** Representative 3D reconstruction of subchondral trabecular bone in the proximal tibia across groups. **(C)** Quantitative analysis of microstructural parameters in proximal tibial trabecular bone among groups. Data are presented as mean ± SD (n = 10 per group). **(D)** TRAP staining of monocytes from RA patients following osteoclast differentiation (n = 3). Representative images from one patient are shown. **(E)** Osteoclast quantification and expression analysis of osteoclast-related markers. Data are expressed as mean ± SD. **(C,E)** One-way ANOVA with Tukey’s multiple comparison. *p < 0.05, **p < 0.01, ***p < 0.001, n.s. not significant. BV/TV, bone volume/tissue volume; Tb.N, trabecular number; Tb.Sp, trabecular separation; Tb.Th, trabecular thickness; M, M-CSF; R, RANKL.

Previous studies indicate that MTX inhibits osteoclast differentiation from peripheral blood-derived mononuclear cells in RA patients (Kanagawa et al., 2016; Teng et al., 2024), but whether NTZ has a similar effect was unknown. We therefore assessed the impact of NTZ on osteoclast differentiation using mononuclear cells derived from a limited cohort of RA patients. NTZ dose-dependently inhibited osteoclast formation *in vitro* (Figure 2D), and this inhibitory effect was further corroborated by reduced osteoclast counts and diminished marker expression (Figure 2E). Taken together, these findings suggest that the dual inhibitory effect of MTX and NTZ on osteoclastogenesis is consistent with the bone-protective effects observed in the combination therapy, although further validation in a larger patient cohort is warranted.

### MTX-NTZ combination reduced the expression of inflammatory cytokines

TNF-α and IL-6 are major culprits that orchestrate synovial inflammation in RA. We then measured the levels of TNF-α, IL-6 and the downstream protein pSTAT3 (Tyr705) in joint synovial tissues. As shown in Figure 3A, their levels were much higher in the CIA group than in the control group. All treatments (NTZ, MTX, and their combination) reduced these levels, with the combined treatment showing the strongest effect (Figure 3B). These results indicate that the reduction of synovial inflammation by MTX-NTZ combination therapy in CIA is due to the inhibition of TNF-α and IL-6 expression and their downstream signaling pathways in synovial tissue.

**FIGURE 3:**
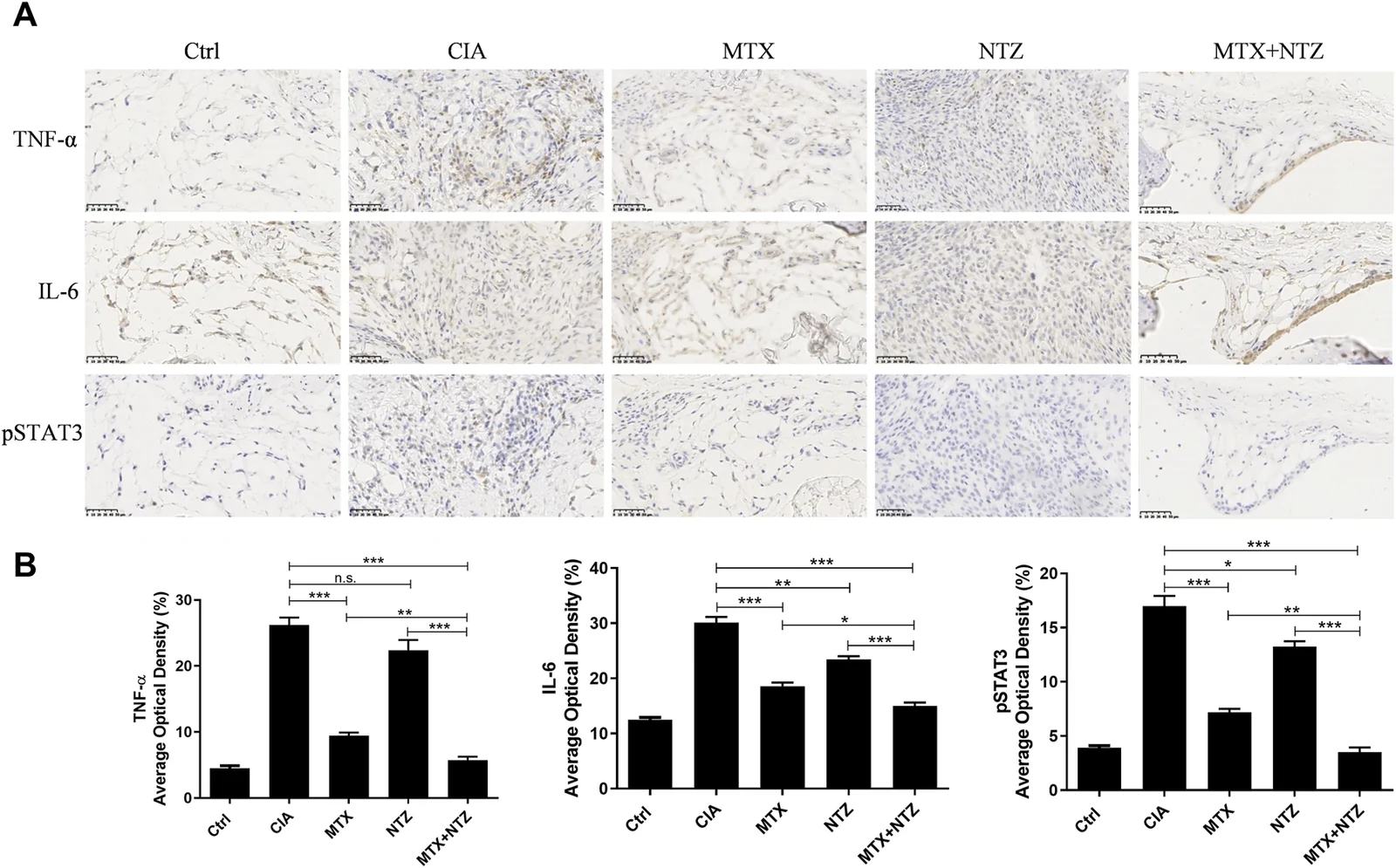
Combined treatment with NTZ and MTX decreased inflammatory cytokine expression *in vivo*. **(A)** Joint tissue sections immunostained for TNF-α, IL-6, and p-STAT3 (Tyr705). Scale bar = 50 µm. Tissue from normal mice served as the control (n = 3). **(B)** Quantification of the average optical density for TNF-α, IL-6, and p-STAT3 (Tyr705) was performed using ImageJ. Values represent mean ± SD. One-way ANOVA with Tukey’s multiple comparison. *p < 0.05, **p < 0.01, ***p < 0.001, n.s. not significant.

### NTZ suppressed inflammatory cytokine secretion and Th17 cell differentiation from immune cells in mice

Our previous work demonstrated that NTZ alleviates synovitis by modulating the biological functions of RA-FLS (Li et al., 2024). Its impact on immune cells, however, remained unexplored. To investigate this, we treated murine splenocytes *in vitro* with NTZ and measured LPS-induced inflammatory cytokine secretion. LPS robustly stimulated cytokines release, whereas NTZ dose-dependently suppressed the production of TNF-α, IL-6 and IL-1β (Figure 4A). NTZ also significantly inhibited B cell-derived IgG production (Figure 4B). We next assessed Th17 differentiation by isolating CD4^+^ naïve T cells and culturing them under Th17-polarizing conditions with NTZ. NTZ dose-dependently inhibited Th17 cell differentiation (Figures 4C,D). Mechanistically, NTZ (40 µM) markedly attenuated STAT3 phosphorylation at Tyr705 induced by IL-6 (Figures 4E,F) or anti-CD3/CD28 antibodies (Figures 4G,H) in CD4^+^ T cells. Moreover, the CIA model provided additional *in vivo* validation that MTX-NTZ combination was more effective than either agent alone at suppressing Th17 cell differentiation (Figure 4I).

**FIGURE 4:**
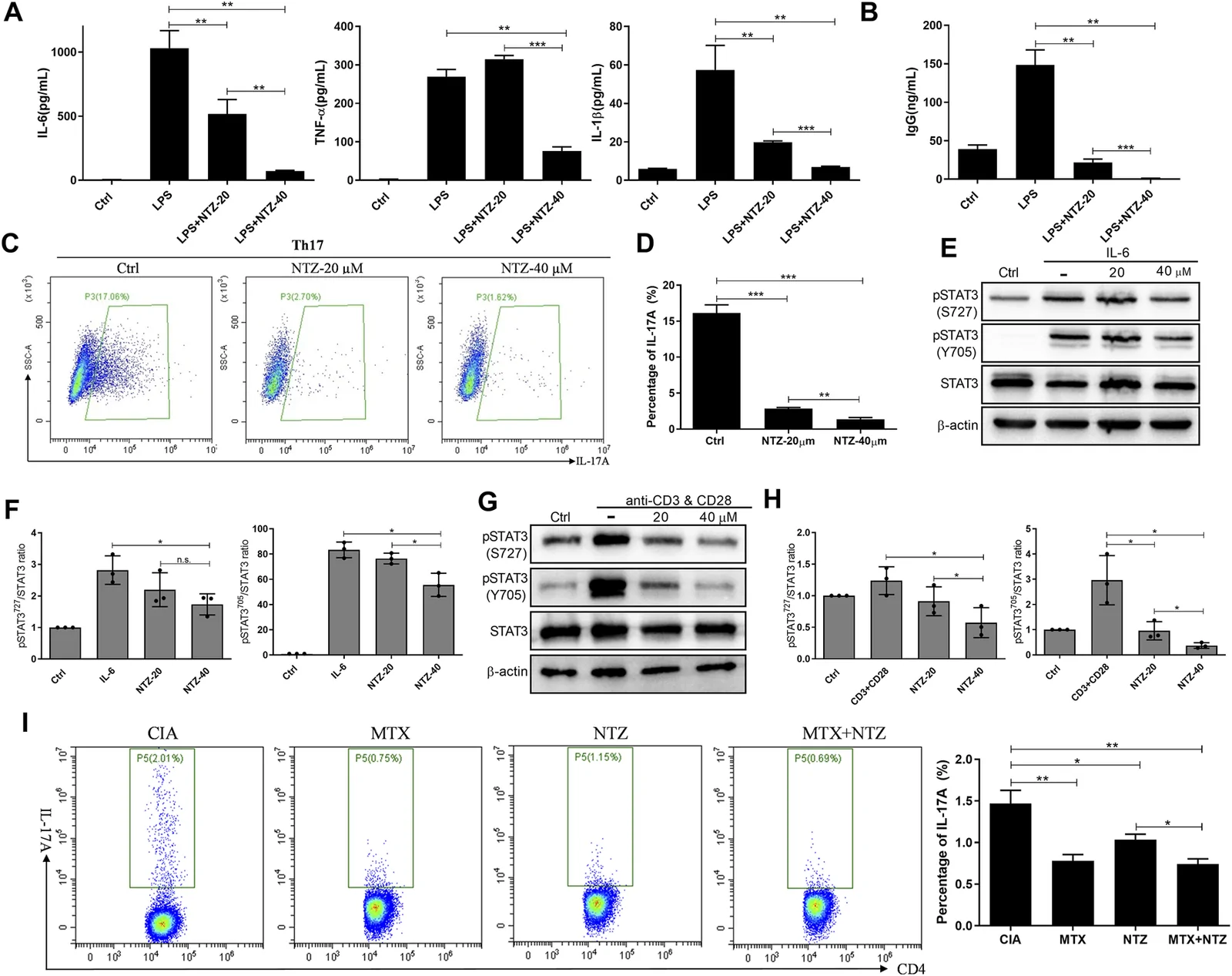
NTZ attenuated inflammatory cytokines production and Th17 cell differentiation by suppressing pSTAT3 signaling activation. **(A,B)** Mouse splenocytes were pretreated with NTZ (20 or 40 μM) for 2 h and then either left untreated (Ctrl) or stimulated with LPS (200 ng/mL) for 3 days. The levels of cytokines and IgG in the supernatant were quantified by ELISA. **(C)** Splenic CD4 T cells were incubated with NTZ (20 or 40 μM) for 2 h, followed by a 3-day culture in Th17-polarizing medium. Cells were then restimulated with PMA and ionomycin in the presence of Brefeldin A for 4 h, and the frequency of IL-17A positive cells within the CD4 population was determined by flow cytometry. **(D)** Data are presented as the percentage of positive cells. **(E–G)** CD4 T cells from mouse spleens were pretreated with NTZ (20 or 40 μM) for 2 h and subsequently exposed to IL-6 (10 ng/mL) for 30 min or anti-CD3/anti-CD28 antibodies for 12 h. Phosphorylation of STAT3 at Tyr705 or Ser727 was analyzed by western blot. The relative ratios of pSTAT3 to STAT3 were quantified by ImageJ. Results are representative of three independent experiments. **(I)** Splenocytes isolated from CIA mice 72 days after primary immunization were *ex vivo* stimulated with PMA and ionomycin in the presence of Brefeldin A for 4 h, and the percentage of IL-17A positive cells was evaluated by flow cytometry (n = 5). Error bars represent mean ± SD. **(A,B,D,H,)** One-way ANOVA with Tukey’s multiple comparison. *p < 0.05, **p < 0.01, ***p < 0.001, n.s. not significant.

### NTZ inhibits inflammatory cytokine production in human PBMCs by interfering with STAT3 phosphorylation

We next performed a preliminary assessment of NTZ on cytokine production and Th17 differentiation using PBMCs isolated from a small number of healthy donors and RA patients. In anti-CD3-activated healthy donor PBMCs, NTZ treatment tended to reduce the secretion of IL-6 and IL-17A (Figure 5A). In RA patient-derived PBMCs, a similar trend was observed, although given the limited sample size, these results should be interpreted as exploratory (Figure 5B). NTZ also appeared to inhibit Th17 cell differentiation in PBMCs from both sources (Figure 5C). At the molecular level, western blotting revealed that NTZ attenuated anti-CD3-triggered STAT3 phosphorylation, particularly at the Tyr705 residue, in the available samples (Figures 5D,E). Collectively, these preliminary findings suggest that NTZ may exert modulatory effects on Th17-associated responses in both healthy individuals and RA patients; however, the small sample size precludes definitive conclusions, and validation in larger cohorts is essential.

**FIGURE 5:**
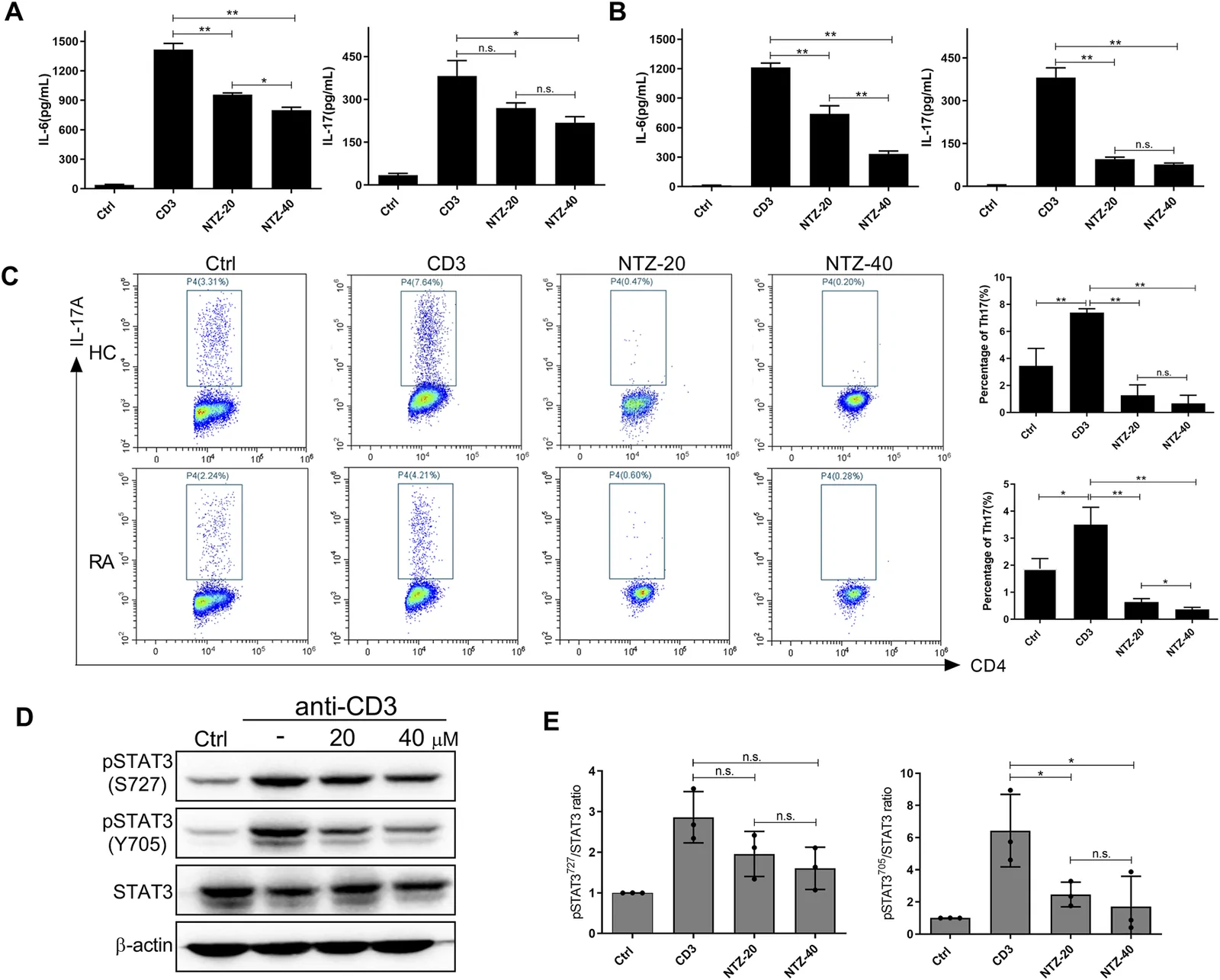
NTZ suppressed inflammatory cytokine secretion in human cells via inhibition of STAT3 phosphorylation. **(A,B)** PBMCs from healthy donors (A, n = 3) and RA patients (B, n = 3) were incubated with NTZ or vehicle for 2 h, followed by no treatment (Ctrl) or stimulation with anti-CD3 antibody for 3 days. Concentrations of IL-6 and IL-17A in the supernatant were measured by ELISA. **(C)** Using the same treatment as in A, PBMCs from healthy individuals (n = 3) and RA patients (n = 3) were restimulated for 4 h with PMA and ionomycin in the presence of Brefeldin A to assess intracellular IL-17A. The proportion of IL-17A + CD4 + T cells was determined via flow cytometry, with results shown as percentages of positive cells. **(D,E)** Healthy donor PBMCs were pretreated with NTZ or vehicle for 2 h and then either left unstimulated (Ctrl) or activated with anti-CD3 antibody for 12 h. STAT3 phosphorylation at Tyr705 and Ser727 was evaluated by western blot, and band intensities were quantified using ImageJ to calculate the pSTAT3/STAT3 ratio. All data are derived from three independent experiments. **(A–C,E)** One-way ANOVA with Tukey’s multiple comparison. *p < 0.05, **p < 0.01, n.s. not significant. HC, Healthy control; RA, Rheumatoid arthritis.

## Discussion

In recent years, progress in elucidating the pathogenesis of RA has facilitated the development of diverse therapeutic agents targeting soluble mediators, cytokine receptors, and intracellular signaling pathways. These treatments have been applied in clinical practice with demonstrated benefits. Nevertheless, approximately 5%–20% of RA patients are classified as having difficult-to-treat RA according to the EULAR definition (David et al., 2024), whereas about 30%–40% of patients exhibit an inadequate response to MTX monotherapy (Duquesne et al., 2023). There is a pressing need to identify new strategies to curb disease progression in RA (Khokhar et al., 2024). However, the development of novel drugs not only requires substantial investment but is also unlikely to bridge this therapeutic gap in the short term. Consequently, drug repurposing has emerged as a promising alternative strategy. Our previous work revealed the therapeutic potential of NTZ for bone resorptive diseases, such as osteoporosis and RA (Li et al., 2021; Li et al., 2024). Although these studies identified osteoclasts and RA-FLS as the primary targets, the effects on classical immune cells and the potential synergy of an MTX-NTZ combination remain unexplored.

In this study, we assessed the protective potential of combining NTZ with MTX against CIA. The two agents acted simultaneously to ameliorate CIA, producing therapeutic outcomes superior to those achieved by monotherapy with either drug. At the mechanistic level, NTZ markedly reduced the *in vitro* secretion of key inflammatory cytokines, namely, IL-6, IL-1β, IL-17A and TNF-α, in immune cells. Furthermore, NTZ effectively suppressed the differentiation of Th17 cells and osteoclasts in both murine and human cellular systems (Figure 6), and these effects collectively contributed to the improvement in arthritis score in the CIA model.

**FIGURE 6:**
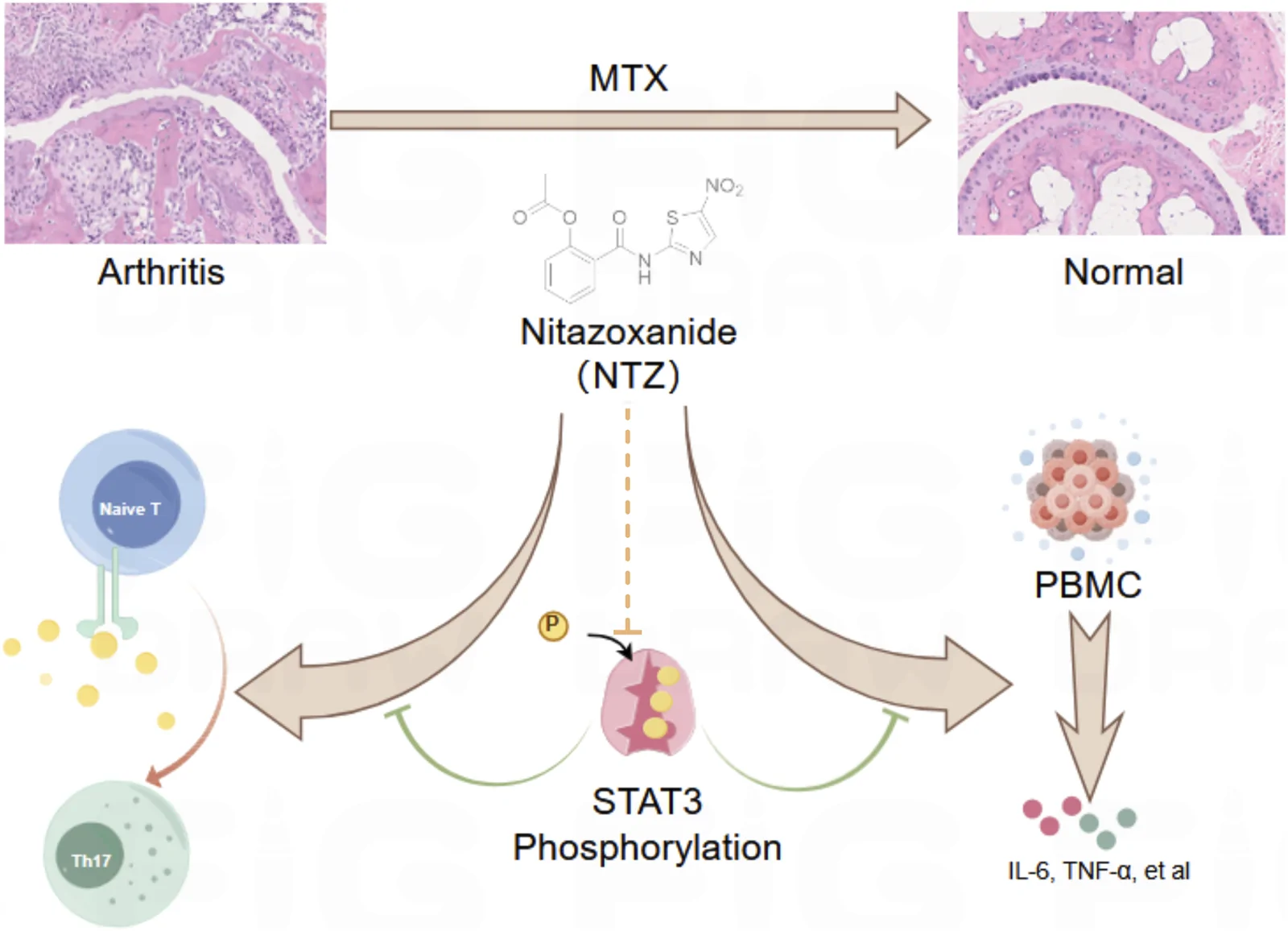
Proposed mechanism of NTZ alleviates CIA. The dashed arrow represents a potential regulatory effect based on current observations, direct binding partners require further biochemical validation.

MTX has served as a cornerstone in the management of RA for decades and continues to be a first-line therapy globally (Zhao et al., 2022). While MTX can effectively alleviate clinical symptoms in many RA patients, it seldom induces complete remission of the disease. Notably, adverse events occur in approximately 30%–45% of individuals undergoing MTX therapy, which may include hepatotoxicity, gastrointestinal disturbances, renal dysfunction, pancreatic cancer, and other drug-related complications (Huang et al., 2020). This underscores the need to refine current single-target treatment approaches and develop multi-target therapeutic strategies for RA. In clinical practice, MTX is increasingly used in combination with biologics as well as with other synthetic drugs, natural products, or peptides (Nakayama et al., 2024; Hao et al., 2017). Our experimental findings demonstrate that the combination of NTZ and MTX more effectively suppressed the progression of arthritis compared to monotherapy with either agent. These results suggest that NTZ has the potential for combination therapy with MTX in the treatment of RA.

This study demonstrates a consistent anti-inflammatory effect of NTZ across experimental models. *In vitro*, NTZ reduced the production of IL-6, TNF-α and IL-1β in murine splenocytes, as well as IL-6 and IL-17 in human PBMCs. This anti-inflammatory effect was further confirmed *in vivo*, where NTZ treatment significantly lowered TNF-α and IL-6 levels in the synovium of CIA mice. Additionally, NTZ dose-dependently inhibited the differentiation of pathogenic Th17 cells *in vitro*. Correspondingly, in the CIA mouse model, NTZ administration markedly suppressed Th17 cell generation, an effect that was enhanced in combination with MTX. The consistency of these observations across diverse experimental settings underscores the robustness of our findings. Nevertheless, it is important to acknowledge that data derived from RA patients may be influenced by concomitant background immunosuppressive therapies, which could serve as a potential confounding factor. This consideration highlights the need for future validation in larger cohorts of treatment-naïve RA patients.

Mechanistically, NTZ treatment markedly suppressed the STAT3 signaling pathway in both mouse immune cells and human PBMCs, regardless of stimulation with IL-6 or anti-CD3 antibody. This inhibition was primarily evidenced by reduced STAT3 phosphorylation, with a more pronounced effect at tyrosine 705 (Y705) than at serine 727 (S727). Such a pattern is consistent with the previously reported inhibitory effect of NTZ on TNF-α–activated JAK2/STAT3 in RA-FLS (Li et al., 2024). While the precise binding mechanism of NTZ to STAT3 warrants further investigation, our findings prompt a speculative hypothesis regarding its functional consequences. It is well established that Y705 phosphorylation is essential for canonical STAT3 nuclear translocation and subsequent inflammatory gene expression, whereas S727 phosphorylation facilitates mitochondrial translocation of STAT3 and supports cell proliferation and mitochondrial uncoupling effects (Peron et al., 2021; Aksu et al., 2026). Based on the observed differential phosphorylation sensitivity, we tentatively suggest that NTZ may preferentially target the canonical Y705-driven transcriptional pathway, with a milder effect on S727-associated mitochondrial functions. However, we acknowledge that this interpretation remains speculative in the absence of direct evidence from nuclear translocation or mitochondrial STAT3 activity assays. Accordingly, we regard this differential effect as a preliminary observation that may guide future structure–activity relationship studies and functional investigations.

This study has several limitations. First, the number of RA patients included is small and all are female, which may introduce selection bias. Second, the cross-sectional design limits our ability to assess temporal changes in the effects of NTZ on osteoclastogenesis and cytokine secretion. Therefore, prospective evaluations of the effects of NTZ in RA patients of both sexes and with varying disease statuses are necessary. Third, the absence of a formal power calculation prior to the animal experiments represents a methodological limitation. While the animal cohort size used in the present study is consistent with the conventional design for the CIA model, we acknowledge that the current sample size may not have been adequately powered to detect smaller effect sizes, should they exist. Fourth, the absence of data on NTZ–STAT3 binding kinetics, pharmacokinetics, and toxicology collectively limits both the mechanistic interpretation of our findings and their translational potential. In addition, whether the moderate inhibitory effect of NTZ on STAT3 S727 phosphorylation contributes to the maintenance of its mitochondrial uncoupling function remains to be elucidated in future studies. Finally, this study only assessed the effect of NTZ on Th17 cell differentiation, without evaluating the Th1 subset, which to some extent weakens the comprehensive interpretation of NTZ’s impact on immune cell function.

In summary, this study shows that NTZ, either as a monotherapy or in combination with MTX, effectively attenuates experimental autoimmune arthritis by regulating key inflammatory cytokines and limiting Th17 cell expansion, as observed in CIA models and RA patients. Beyond its immunomodulatory actions, NTZ also suppresses osteoclastogenesis in cells derived from RA patients. These data highlight its translational potential as a novel treatment strategy for RA.

## Data Availability

The original contributions presented in the study are included in the article/Supplementary Material, further inquiries can be directed to the corresponding authors.

## Supplementary material

SUPPLEMENTARY TABLE 1 The reagent or resource utilized in flow cytometry, CIA induction, osteoclast and Th17 cell differentiation.

SUPPLEMENTARY TABLE 2 Clinical data associated with *in vitro* experiments.
